# Practice of code of ethics and associated factors among health professionals in Central Gondar Zone public hospitals, Northwest Ethiopia, 2021: a mixed-method study design

**DOI:** 10.1186/s12910-022-00807-7

**Published:** 2022-07-01

**Authors:** Gebreyohannes Yeshineh, Amsalu Feleke, Chalie Tadie, Asebe Hagos, Wubshet Debebe, Getachew Teshale, Lake Yazachew

**Affiliations:** 1 Tegedie Woreda Health Office, Tegedie, Ethiopia; 2 Department of Health Systems and Policy, Institute of Public Health, College of Medicine and Health Sciences, University of Gondar, P. O. Box 196, Gondar, Ethiopia

**Keywords:** Code of ethics, Health professionals, Central Gondar Zone, Ethiopia

## Abstract

**Background:**

Ethics is the science of moral and ethical rules recognised in human life and attempts to verify what is morally right and wrong. Healthcare ethics is seen as an integrated part of the daily activities of health facilities. Healthcare professionals’ standardisation and uniformity in healthcare ethics are urgent and basic requirements. Therefore, this study aimed to assess the practice of the code of ethics and associated factors among health professionals in Central Gondar Zone public hospitals, Northwest Ethiopia, 2021.

**Methods:**

A facility-based cross-sectional study design with a mixed method was conducted on 631 health professionals from Central Gondar Zone public hospitals. For the quantitative part, pre-tested self-administered questionnaires were used, and for the qualitative part, key informant interviews with a semi-structured questionnaire were used. Variables with a *p* value of < 0.2 in binary logistic regression entered into a multivariable logistic regression, then *p* value < 0.05 and AOR were used to declare statistically significant variables in quantitative data. A thematic content analysis was used for qualitative data analysis.

**Results:**

This study revealed that only 286 (46.7%) health professionals had good practice of the code of ethics. Good ethical knowledge (AOR = 1.95, 95% CI 1.37, 2.77), favourable attitude (AOR = 1.55, 95% CI 1.11, 2.16), and satisfaction of health professionals with their jobs (AOR = 1.45, 95% CI (1.04, 2.04) were significantly associated with the practice of health care ethics.

**Conclusions:**

Health professionals' overall level of practice of health care ethics in the Central Gondar Zone public hospitals was poor. This necessitates practical training, ongoing follow-up, availing of necessary medical equipment, a smooth working environment, and modification of the recognition system for health professionals.

## Background

Ethics is the science of moral and ethical rules recognized in human life [1], which attempts to verify what is morally right and wrong in human action. Ethics includes not only what should be done but also what must be done in a compassionate, respectful and caring manner [2, 3]. The Geneva Declaration of the World Medical Association and International Code of Medical Ethics announced that physicians are required to act in the patients’ best interests which strengthens the effect of identifying their physical and psychological conditions during healthcare provision [4].

However, ethical dilemmas in the healthcare setting are common. Professionals who are working in health care often deal with ethical issues related to end-of-life care, resuscitation, consent, competence, care and treatment decisions, and overall organisational healthcare management [5, 6]. Healthcare ethics has been seen as an integrated part of health care workers' daily activities in the health facilities [6].

Healthcare ethics is a sensitive framework considered as a part of professionalism for healthcare providers. The poor practices of healthcare ethics, unsatisfactory management, and handling of cases paralysed the service and hampered the service quality. Medical students, residents and nurses have been observed and reported unethical actions in different settings [7].

Although health care ethical principles are universally accepted by various countries, each country can make certain modifications, frames, and specific interpretations consistent with their existing philosophy, spiritual views, and principles of health care practice in the health system [8].

In Ethiopia, the professionals' Code of Ethics has been launched through Regulation No. 299/2013 to recognise health professionals' ethics for the safety of clients. According to this regulation, the Federal Health Professionals Ethics Committee (FHPEC) was established in 2014. This committee with Ethiopian Food, Medicine and Health Care Administration and Control Authority (EFMHACA) had responsibilities to identify, analyse, and develop different measures on complaints of health services, incompetency, and ethical issues of health professionals [9].

The increased community awareness about health professionals' responsibilities and mandates leads to increasing complaints against health professionals. The changing health professional-client relations and healthcare marketing have affected the practice of healthcare services [10].

Improper practice of health care ethics, poor management, and solution to health care service errors threaten a patient-provider relationship and the quality of health service delivery [7, 11].

In a study conducted in Nepal, among 1,600 resident medical doctors and front-line health care leaders, 95% reported that they had routinely dealt with unethical and disruptive behaviours including, insults, yelling, disrespect, abuse, and refusal to carry out responsibilities. These misbehaviours were observed on nurses, physicians, and health facility administrators [12]. Poor knowledge of health care ethics by health professionals leads to many unethical practices in their daily/routine activities [13]. Recently raised in a grievance against healthcare practitioners is an issue of immediate concern [10, 14].

Health professionals who were unsatisfied with their job and the working environment performs unethical activities during their daily work [15]. Despite all codes and regulations, reports of unethical behaviour of health professionals are standard [1]. Shortage of useful health care information, inadequate supervision, and poor compliance accepting mechanisms of the healthcare services from users are factors for unethical health care practices [16]. There is lack of proper and detailed knowledge of healthcare ethics among health professionals that leads to poor practice of healthcare ethics [10]. In Ethiopia, nurses are dissatisfied with their professions due to low monthly salary, lack of incentives and refreshment trainings, workload, lack of respect from society and colleagues, and insecured life insurance. All these factors leads to poor adherence to ethical healthcare practices [17].

Despite their differences in education, professional responsibilities, and perceived medical norms and conducts, physicians and nurses are the critical pillars of healthcare delivery. There for, standardisation and uniformity in health care ethics among all healthcare professionals is an urgent and essential requirement [18–20].

In practice, intentionally or unintentionally, most health care workers, especially front-line providers have committed unethical and unacceptable actions [6, 21, 22]. Thus, scientific evidences and recommendations are critical to solve such problems. Generally, there is limited information regarding the practice of the code of ethics while health professionals provide health care services to clients. The study would provide information about the level of the code of ethics implementation and associated factors among health professionals working in public hospitals of Central Gondar Zone and provide recommendations to track the practice of healthcare ethics and ensure appropriate health service utilisation.

## Methods

### Study design, setting and participants

Facility-based cross-sectional quantitative study supported by a qualitative approach was conducted from March to April 2021 to assess the practice of the code of ethics among health professionals working in public hospitals in the Central Gondar Zone. The Central Gondar Zone is located 738 km from Addis Ababa, the capital city of Ethiopia, and 180 km away from the regional capital city, Bahir Dar. It had 94 health centers, 154 health posts, ten hospitals and within these institutions 430 medical doctors, and 772 nurses served the community. The hospitals served about 7 million people as diagnostic, treatment, and teaching centers.

For both quantitative and qualitative data, all medical doctors and nurses working in public hospitals of Central Gondar Zone were the source population. While all medical doctors and nurses working in the selected public hospitals of Central Gondar Zone were the study population. But those having less than six months of work experience were excluded from this study.

### Sample size and sampling technique

The sample size for the practice of code of ethics was determined by using a single population proportion formula with a 95% confidence interval (CI), 45.6% population proportion (from a study result in Bale Zone Oromia region) [23], 5% margin of error, 1.5 design effect and 10% non-response rate n = $\frac{(\mathrm{Z}\upalpha/2)2\mathrm{ p }(1-\mathrm{p}) }{\mathrm{d}2}=\frac{(1.96)2*0.456(1-.456) }{0.0025 }$

n = 382*1.5= 573 with 10% non-response rate it becomes 631.

**Fig. 1:**
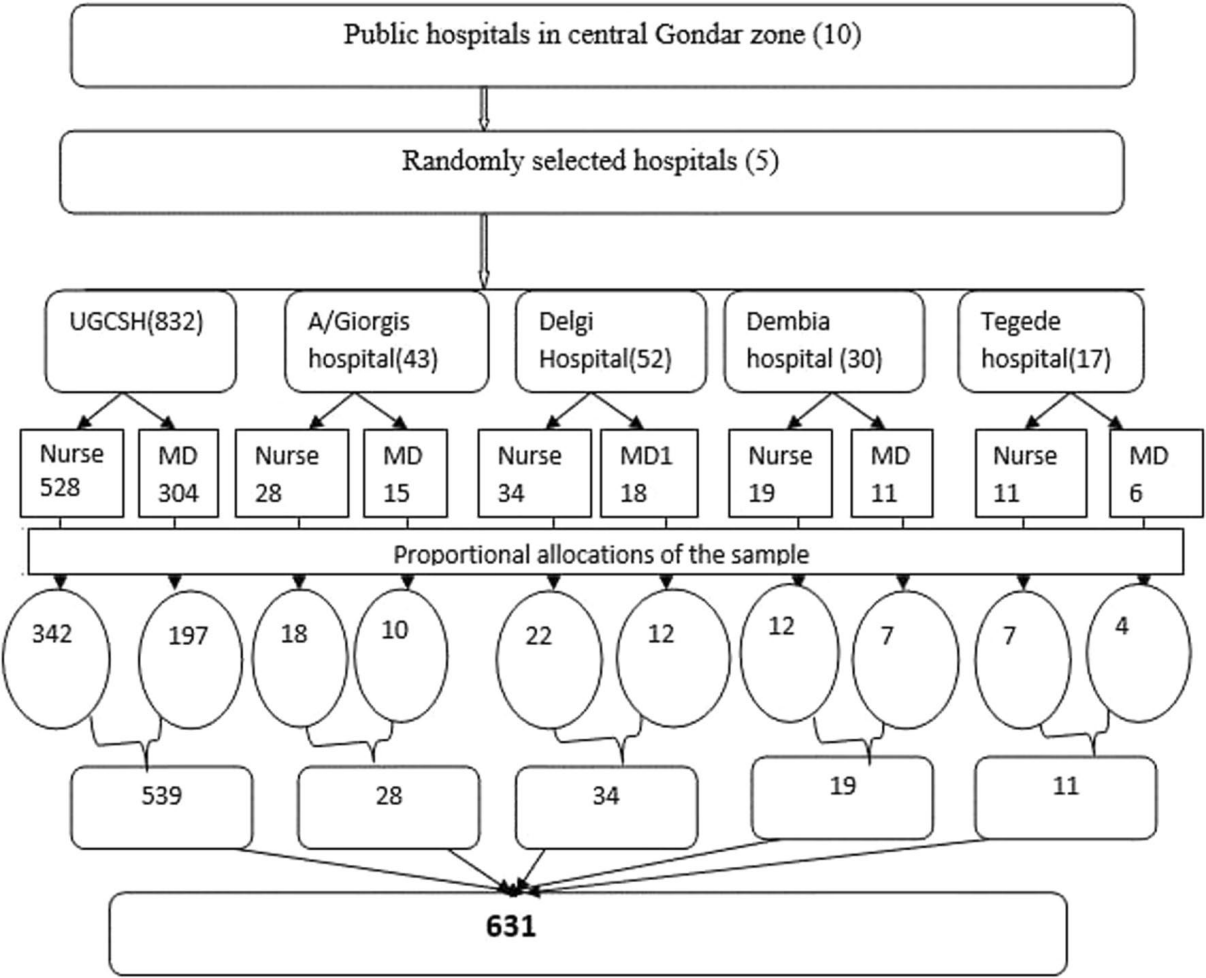
Schematic sampling procedure of health professionals in Central Gondar Zone, Ethiopia, 2021. Key: UGCSH; University of Gondar Comprehensive and Specialized Hospital, A/Giwergis; Amba Giworgis, MD; Medical Doctors

A multistage sampling technique was used to get the study participants. Simple random sampling method was applied to select the first five (50%) hospitals from the total hospitals in the Zone [24]. Second, the list of health professionals (nurses and medical doctors) was identified from human resource registration at each selected hospital to consider a sampling frame. Then, the sample size was proportionally allocated to each selected hospital based on the number of available nurses and medical doctors. Simple random sampling (Open Epi Random Program version 3) was used to select from each hospital's nurse and medical doctor professionals (Fig. 1).

For qualitative data, the sample size is determined by information saturation and the samples were selected purposively based on their work experience and position in the hospitals. Based on these criteria, medical directors, metron nurses, and case managers were included for KII.

### Variables and measurement

The practice of the code of health care ethics was the dependent variable. It was measured by using 26 standardised item questions with a 5-point Likert scale (Table 1). The response related to the practice of the code of ethics was ategorized as good practice when the score was greater or equal to 75%, and poor practice if it is less than 75% [8].

**Table 1:** Practice of code of ethics-related questions in public hospitals of Central Gondar Zone Northwest, Ethiopia, 2021

Variable	Response category
How often you have interest in learning healthcare ethics?	5-Always 4-Very often 3-Some times 2-Rarely 1-Never
How often do you obtain informed consent from a patient before rendering a service?	
How often do you think health professionals must serve hard to reach areas and underserved populations?	
How often consent is required only for surgeries, not for tests and medicines?	
How often children should never be treated without the consent of their parents?	
How often health professionals should do their best irrespective of the Patient's opinion?	
How often health professionals should refuse to treat a violent patient?	
How often do you respect patient confidentiality, privacy, choices and dignity?	
How often close relatives should always be told about a patient's condition?	
How often ethical conduct is only important to avoid legal Action?	
How often do you provide health service for your benefit that does not serve the needs of your Patient?	
How often do you work with or give any professional support to other health professionals not licensed by appropriate organ?	
How often do you render the same Level of care to your clients in overtime and regular Practice?	
How often do you provide any preferential treatment to a client/patient by considering the relationship established with you in other health institution where you work?	
How often do you use secret remedies to treat a patient?	
How often do you use an apparatus or health technology or intervention which is proved upon investigation to be capable of fulfilling the claims made regarding it?	
How often do you refuse on the ground of your personal belief to provide services such as contraceptives, legal abortion and blood transfusion?	
How often do you sign and write your name on official documents relating to patient care such as laboratory and other diagnostic requests and results, prescriptions, certificates, patient records and other reports?	
How often writing when it hasn't been done, is acceptable because it is important for documentation?	
How often do you issue genuine and complete sick leave or certificate of illness?	
How often do you prescribe medicine or formulations about which you do not know about its composition and pharmacological Action?	
How often do you prescribe medicine not registered on the National Medicine List without compelling reason?	
How often if a patient wishes to die, he or she should be assisted? in doing so no matter what their illness?	
How often do you report impairment in other health professionals to the appropriate organization if you are aware of it?	
How often do you report your impairment to the appropriate organ if you are aware of it?	
How often do you report any unprofessional/unethical conduct of another health professional to the appropriate organ?	

Whereas, Socio-demographic factors (age, sex, religion, occupation, educational level, monthly income, and work experience), Institutional related factors (training, type of health facility, availability of policy, access to guidelines), Individual related factors (knowledge, attitude, work experience, and Job satisfaction), and professional ethics-related factors (ethical dilemma, ethical problem) were the independent variables.

*Knowledge about code of ethics*: assessed by nine-item yes or no questions related to practice of code of ethics (Table 2). Participants who answered greater or equal to 75% were knowledgeable, while less than 75% were not knowledgeable [8].

**Table 2:** knowledge-related questions of health professionals regarding the Practice of health care ethics in public hospitals in Central Gondar Zone, Northwest, Ethiopia, 2021

Items	Response category
1. Understanding that there is a code of ethics	1. Yes 2. No
2. If yes, Is professional ethics an important subject	
3. Right of the Patient should always be recognized	
4. Disclosure of medical report to 3^rd^ party is important	
5. The establishment of a friendly relationship is important	
6. The patient has the right to know about his/her problem	
7. Being unethical leads to legal Action	
8. Patients who want to die should be assisted	
9. Disclosing medical error only if it caused major harm	

*Attitude towards code of ethics*: assessed by 14-item questions with a 5-Likert scale response. Among the 14 item questions related to the attitude of professional ethics, those participants who answered greater or equal to 75% of the questions had favourable attitudes, otherwise unfavourable attitudes (Table 3) [8].

**Table 3:** Attitudes related questions of healthcare professionals towards health care ethics in public hospitals in Central Gondar Zone, Northwest, Ethiopia, 2021

Questions	Response categories
The only way to avoid legal Action is to conduct yourself ethically	1. Strongly agree 2. Agree 3. Neutral 4. Disagree 5. Strongly disagree
Patient's wishes must always adhere	
Health professionals should do what is best irrespective of the patient's option	
The patient should always be told if something is wrong	
Confidential information may only be disclosed if the patient gives explicit consent	
In modern care, confidentiality is inapplicable and should be abandoned	
Close relatives should always be informed of the patient's status	
Patients need to consent only for operations but not for tests or medication	
Always seek the consent of the child's parents before treating the child	
Violence should not be tolerated in the treatment of patients by health professionals	
It is correct to treat social clients first before other patients	
Reporting co-worker misconduct is correct	
Patients who refuse treatments due to beliefs should be instructed to find another health professionals	
Health professionals should resolve conflicts with other health care providers	

*Job satisfaction*: assessed by nine-item questions related to job satisfaction. Participants who answered greater or equal to 50% considered satisfied; otherwise, not satisfied [25].

*Health professionals*: those who graduated from a known university or College as medical doctors or nurses.

*Ethical dilemma*: hampering smooth decision-making and poor working relations among staff. Resource constraints, poor attitude of some staff towards work, conflicts among ethical codes, religious beliefs, and personal values.

### Data collection

Both quantitative and qualitative data were collected in parallel. Two BSc nurses and one health officer participated in the quantitative data collection and as supervisor respectively. The data collectors and the supervisor were well experienced in data collection activities. The principal investigator collected the qualitative data.

### Data quality control

Two days of training were given to data collectors about the primary data collection techniques. A pre-test was also conducted on 5% of participants at a neighbouring hospital. During data collection, the principal investigator and supervisor checked the questionnaire for its completeness daily. The principal investigator transcribed and checked the consistency of the information with the initials. Key informant interviews (KIIs) were conducted until the saturation of information.

### Data processing and analysis

Data was entered into Epi-Info version 7.0 and exported to SPSS version 20 for analysis. Data were cleaned and checked for errors and missing observations to ensure data accuracy and consistency. For the qualitative study, the tape-recorded audio and notes from interviews were used and transcribed manually. Finally, the data was read to identify key themes and synthesised thematically.

A variety of descriptive statistics were presented by using tables and narrations. Binary logistic regression models were used. Model fitness and multi co-linearity were checked by using the Hosmer and Lemeshow test and variance inflation factor respectively. Finally, in multivariable logistic analysis with adjusted Odds Ratio (AOR), 95%CI and *p* value ≤ 0.05 were declared statistically significant variables.

## Results

### Socio-demographic characteristics of health professionals

In this study, 613 health professionals with a response rate of 97.15% were participated. Among the study participants 332 (54.2%) were males and 376 (61.9%) of them were 30–39 years old. The majority (88.4%) of the respondents had a first degree and 226 (36.9%) of them had 4–8 years of work experience (Table 4).

**Table 4:** Socio-demographic characteristics of health professionals (n = 613)

Variable	Category	Frequency (n)	Percentage (%)
Sex	Male	332	54.2
	Female	281	45.8
Religion	Orthodox	483	78.8
	Muslim	88	14.4
	Protestant	39	6.4
	Catholic	3	5
Age	20–29 years	187	30.5
	30–39 years	376	61.9
	39–50 years	50	8.2
Marital status	Married	286	46.7
	Single	286	46.7
	Divorce	38	6.2
	Widowed	2	0.3
	Separated	1	0.1
Level of education	Diploma	31	5.1
	First degree	542	88.4
	Masters	15	2.4
	Specialist	21	3.4
	Subspecialist	4	0.7
Type of Health professionals	Medical doctors	223	36.4
	Nurses	390	63.6
Work experience in years	< 4	193	31.5
	4–8	226	36.9%
	> 8	194	31.5%
Monthly income	3000–5000	46	7.5%
	5000–7000	163	26.6%
	7000–26,000	404	65.9%

### Profession related factors

Among the study participants, 515 (84%) were happy with their current profession, and 549 (89.6%) chose their current profession by when they joined the university. Most study participants (80.9%) were ready to recommend others to pursue their current professions. One hundred eighty-eight (30.7%) of the participants did not know about the existence of the ethics committee in their hospitals. Among those who knew about the existence of an ethics committee in the hospital, 207 (48.7%) replied positive correction measure was the main task of the committee, 11 (2.6%) and 173 (40.7%) of the respondents replied that training and advice as duties of the ethics committee, respectively. The remaining 34 (8%) replied that punishment is the duty of the ethics committee.

Four hundred eighty-six (79.3%) of the participants mentioned college/university as the primary source of information for professional ethics, while the remaining 127(20.7%) health professionals consider training, working organizations, and colloquies were sources of information about professional ethics. Regarding professional ethics delivered as a curriculum course, 507 (82.7%) of the respondents replied that it was not adequate, 97 (15.8%) thought that it was adequate, and the remaining 9 (1.5%) didn't remember the course adequacy.

One hundred forty-seven (24%) of the study participant had been accused of unethical health care issues. Truth-telling was the most common ethical dilemma 229 (62.4%). Among health professionals perceived unethical health care practice 253 (41.3%) were due to insufficient salary. Only 162 (26.4%) hospital administrators communicated professional ethics as a priority issue, and 317 (51.7%) of the respondents said the promotion decision doesn't consider the ethical practice. Four hundred forty-three (72.3%) participants replied that a relationship with managers negatively influences ethical practice, while 172 (28.1%) health professionals frequently entered into conflicts with their colleagues. About 170 (27.7%) of study participants used substances in their lives. Among them, 82 (48.2%) chewed khat, and 49 (28.8%) drank alcohol (Table 5).

**Table 5:** Profession-related characteristics of health professionals in Central Gondar Zone public health Hospitals in 2021 (n = 613)

Variables	Frequency (n)	Percentage (%)
Happiness with current profession		
Yes	515	84
No	98	16
Was it first choice of your profession?		
Yes	549	89.6
No	64	10.4
Advising of others to pursue your profession		
Yes	496	80.9
No	117	19.1
Source of information for code of ethics		
University/college	486	79.3
Other*	127	20.7
Know the presence of ethics committee		
Yes	425	69.3
No	188	30.7
The function of the ethics committee		
Correction measure	207	48.7
Punishment	34	8.0
Advise	173	40.7
Training	11	2.6
Curriculum adequacy in the University/college		
Yes adequate	97	15.8
Not adequate	507	82.7
I don't remember	9	1.5
Facing ethical problems in his/her experience		
Yes	413	67.4
No	200	32.6
Perceived reason for unethical Practice		
Workload	338	38.8
Negligence	66	10.8
Lack of Knowledge	56	9.1
Insufficient salary	253	41.3
Accused regarding an ethical issue		
Yes	147	24
No	466	76
Encountered ethical dilemma		
Yes	367	59.9
No	246	40.1
Common ethical dilemma		
Religious issue	6	1.6
Improper discharge	116	31.6
Truth-telling	229	62.4
End-of-life issue	16	4.4
Promotion decision considering an ethical practice		
Yes	315	51.4
No	298	48.6
Relationships with managers negatively influence ethical Practice		
Yes	443	72.3
No	170	27.7
Administrators communicate ethics as a priority		
Yes	162	26.4
No	451	73.6
Frequent colleagues conflict		
Yes	172	28.1
No	441	71.9
Harassment from Patient		
Yes	279	45.5
No	334	54.5
Receiving a gift from a patient		
Yes	39	6.4
No	574	93.6
Have you ever used substance		
Yes	170	27.7
No	443	72.3
Type of substance		
Alcohol	49	28.8
Khat	82	48.2
Cigarette	22	12.9
Drug	17	10.1
Satisfaction with your job		
Satisfied	317	51.7
Unsatisfied	296	48.3

*Training, mass media, peers

### Knowledge and attitude towards health care ethics

The knowledge and attitude of health professionals towards health care ethics showed that 392 (63.9%) had good knowledge about healthcare ethics, and 319 (52%) had a favourable attitude toward health professional ethics (Table 6).

**Table 6:** Knowledge, Attitude and practice of code of ethics among health professionals in Central Gondar Zone public Hospitals, 2021 (n = 613)

Variables	Frequency	Percentage (%)
Knowledge		
Good	392	63.9
Poor	221	36.1
Attitude		
Favourable	319	52
Un favourable	294	48
Practice		
Good Practice	286	46.7
Poor practice	327	53.3

Key informants also confirmed that even though healthcare professionals knew the code of healthcare ethics, they didn't practice it scientifically.

All health professionals had baseline knowledge about health care ethics during college/university education. But health professionals were not doing what they know scientifically; instead, they exercise traditionally adopted from the environment and perform things negligently.

(30 years old male, medical director in one of the selected hospitals)

Although knowledge is subjective unless surveyed, we believed that all health professionals knew health care ethics before they leave educational institutions and through different job training about code of ethics.

(28 years old female case manager in one hospital)

### Practice towards health care ethics

The degree to which health professionals demonstrate actions consistent with ethical practices in health care delivery was 46.7% (95%CI: 42.7, 50.6) (Table 6). All key informants said that, even if there was a slight improvement in the practice of health care ethics, we had a problem implementing it. The significant gaps related to the practice of code of ethics among health professionals were lack of information, notice and advice, lack of respect for client rights and autonomy, such as not declaring and elucidation the consent well for clients and not taking informed consent continuously, ordering medication and conducting physical examinations without consultation of a patient in most of the time except in surgery. There was also a problem in providing health services in the proper working place and at the required time for some health professionals in hospitals.

Most health care providers have problems with patient consent and information giving regarding the service they gave to the clients. This is the most violated ethical principle. Because when patients go to the service giver, most departments did not take consent from the patients except while they prepared for surgery*.*

(33 years old matron nurse in one selected hospital)

### Institutional related factors

Four hundred twenty-three (69%) study participants were from the specialised hospital. Among the study participants, 573 (93.5%) health professionals knew about the presence of health care ethics-related documents in their hospitals. Study participants confirmed that those who comply with the law and professional standards were 391 (63.8%) and those health professionals who have awareness about the presence of health care ethics were 389 (63.5%). In addition, participants confirmed that health professionals who enacted the hospitals' rules and standards were successful, and 469 (76.5%) of participants obeyed the hospital rules and regulations (Table 7). Key informants' interviews also supported the quantitative findings.

Even though most of our hospital health professionals had a positive attitude to perform, they are also disappointed by political interference of hospital management and lack of medical equipment. Health professionals were not recognised and promoted as per the given responsibility and duties; instead, they were recognised due to the approach with their managers. Relatively those who had long experience in their work were better implementers of principles of health care ethics.

38 years old male, metron nurse in one of the selected hospitals

**Table 7:** Institutional factors for the practice of code of ethics among health professionals (n = 613)

Variables	Categories	Frequencies (n)	Percentage (%)
Type of Hospitals	Specialized	495	80.8
	Primary	118	19.2
Is there health ethics document in the Hospital	Yes	389	63.5
	No	224	36.5
Comply with law and professional standards over other consideration	Yes	391	63.8
	No	222	36.2
The law and ethical code of their professional is the major consideration	Yes	431	70.3
	No	182	29.7
People are expected to strictly follow legal/professional standards	Yes	408	66.6
	No	205	33.4
Decisions that violet any law	Yes	379	61.8
	No	234	38.2
Very important to follow the hospital rule and procedure	Yes	441	71.94
	No	172	28.06
Every health professional is expected to stick by the hospital rule and procedure	Yes	430	70.1
	No	183	29.9
Successful people in the hospital go by the book	Yes	400	65.3
	No	213	34.7
People in this hospital strictly obey the Hospitals policy	Yes	469	76.5
	No	144	23.5

### Factors associated with the practice of code of ethics

Knowledge, attitude, job satisfaction, monthly income, work experience, people's expectation of legal/professional standards, relationship with managers, promotion related to ethical practice, and accused regarding the ethical issue were candidate variables entered into multivariable logistic regression analysis. Among these, knowledge of health care ethics, attitude of health professionals towards health care ethics, and job satisfaction of health professionals were significantly associated with the practice of the code of ethics.

This study showed participants who had good knowledge of the healthcare code of ethics were 1.95 times more likely to exercise practice of healthcare code of ethics (AOR 1.95; 95% CI 1.37, 2.77) compared to those who had poor knowledge of healthcare code of ethics. Similarly, healthcare professionals who had a favourable attitude towards the healthcare code of ethics were 1.55 times more likely to apply a good practice (AOR 1.55; 95% CI 1.11, 2.16) as compared to those who had an unfavourable attitude. Satisfied health professionals with their job were 1.45 times more likely to exercise ethical health care practice (AOR 1.45; 95% CI 1.04, 2.04) than unsatisfied health professionals (Table 8).

**Table 8:** Bivariable and multivariable analyses for factors associated with the practice of code of ethics among health professionals (n = 613)

Variables	Categories	The Practice of code of ethics (n = 613)		COR with 95% CI	AOR with 95% CI
		Good	Poor		
Knowledge	Good	206 (33.6)	186 (30.3)	1.952 [1.392–2.738]	1.951 [1.374–2.770]**
	Poor	80 (13)	141 (23)	1	1
Attitude	Favorable	164 (26.7)	155 (25.3)	1.492 [1.084–2.053]	1.547 [1.106–2.163]*
	Unfavorable	122 (19.9)	172 (28)	1	1
Income (Ethiopian Birr)	< 5000	30 (4.9)	16 (2.6)	2.176 [1.150–4.116]	1.836 [.932–3.615]
	5000–7000	69 (11.3)	94 (15.3)	0.852 [0.590–1.230]	.820 [0.540–1.246]
	> 7000	187 (30.5)	217 (35.4)	1	1
Work experience	< 4 years	104 (17)	89 (14.5)	1.467 [0.983–2.190]	1.483 [.959–2.294]
	4–8 years	96 (15.7)	130 (21.2)	0.927 [0.630–1.366]	0.933 [.600–1.452]
	> 8 years	86 (14)	108 (17.6)	1	1
Job Satisfaction	Satisfied	165 (26.9)	152 (24.7)	1.570 [1.140–2.162]	1.453 [1.037–2.036]*
	Un satisfied	121(19.7)	175 (28.4)	1	1
People Expected Strictly Follow Professional Standard	Yes	205 (33.4)	203 (33.1)	1.546 [1.100–2.173]	1.417 [0.989–2.030]
	No	81 (13.2)	124 (20.2)	1	1
Relationship With Manager Negatively Influenced Practice	Yes	196 (32.0)	247 (40.3%)	0.705 [0.495–1.006]	0.725 [0.491–1.070]
	No	90 (14.7)	80(13.0)	1	1
Promotion Decision Considering Ethical Practice	Yes	146 (23.8)	169 (27.6)	0.975 [0.710–1.339]	1.137 [0.796–1.624]
	No	140 (22.8)	158 (25.8)	1	1
Accused regarding Ethical Issue	Yes	54 (8.8)	93 (15.2)	0.586 [0.400–0858]	0.635 [.426–0.948]
	No	232 (37.8)	234 (38.2)	1	1

*Statistically significant at *p* value < 0.05; **statistically significant at *p* value < 0.01

## Discussion

In this study, the overall practice of health care ethics was poor, 46.7% (95% CI 42.7, 50.6). The qualitative findings also indicated that even if there was a slight improvement in healthcare ethics by health professionals, there were problems in implementing healthcare ethics.

This finding is consistent with the study conducted in Pakistan, Egypt, and Bale zone in Ethiopia that resulted in 50%, 48%, and 45.6% of health professionals having a poor practice of health care ethics [1, 21, 23]. In contrast, the finding of this study was higher than a study conducted in Mekelle, Ethiopia, among nurse professionals [17]. The possible reasons might be due to CRC training, relatively smooth relationships with the hospital management, and a safe working environment [26].

The qualitative findings also indicated that lack of information and counseling, respect for patient rights and freedom, not explaining the consent well to patients and not taking informed consent were the significant problems to practice the code of ethics. Additionally, the study showed a lack of adequate information, communication and counseling, and consideration of client/patient rights and autonomy.

Knowledge about the professional code of ethics is an essential attribute for the practice of health care ethics. This study identified knowledge as one of the factors significantly associated with the practice of health care ethics. Those health care workers with good knowledge about health care ethics were 1.95 times more likely to have good practice of health care ethics compared with health workers with poor knowledge of health care ethics. The finding is supported by studies done in the Bale zone Oromia region, and Addis Ababa [8, 23]. This may be since health professionals who know the code of ethics may understand what is correct and incorrect in implementing health care ethics. The qualitative finding also supported this result. Respondents reported that healthcare professionals with good knowledge of healthcare ethics implemented it better than those who had poor knowledge.

In this study, a favourable attitude towards healthcare ethics was significantly associated with healthcare ethics practice. Practices of code of ethics among health professionals with favourable attitudes were 1.54 times more likely to apply health care ethics than those with unfavourable attitudes towards health care code of ethics. A favourable attitude towards health care ethics improved practice of health care ethics. Facts indicated that people could understand things positively and properly differentiate the possible outcome with those positive attitudes. Similar study findings were reported in Ghana, Gondar town, and north Shewa [22, 26, 27].

Health care professionals satisfied with their job were 1.45 times more likely to apply good health care ethics practice than their counterparts. A similar study was conducted in Gondar comprehensive specialised hospital and western Amhara [25, 28]. This can be explained by the fact that when healthcare professionals are satisfied with their job, health professionals may respect the code of healthcare ethics. Health care professionals' satisfaction with their job may reflect good management practice. Thus, like positive reinforcement healthcare professionals may adhere to the code of healthcare ethics.

Our finding indicates that less than half of health professionals had good ethics practices. This implies that most health care professionals, particularly front-line providers, have engaged in unethical and improper behaviour, whether intentionally or accidentally. Therefore, it is crucial to counter act the problem by working with the health professionals.

### Strengths and limitations

The study acknowledges the following limitations. First, health professionals working in specialised hospitals and primary hospitals were influenced by environmental factors and healthcare infrastructure to apply healthcare ethics. They should be seen separately to know the level of practice based on institutional hierarchy. Second, the findings of this research referred to the front-line health professionals of medical doctors and nurses, so the results can only be generalised to these health professionals and may not reflect the practice of code of ethics by other health professionals like midwives, health officers, laboratory technicians, and pharmacists. Third, there might be a social desirability bias. To minimize it, self-administered questionnaires were employed. Finally, the cross-sectional nature of the study also affects the determination of cause and effect relationships.

## Conclusions

The practice of the code of ethics among health professionals working in public hospitals in the Central Gondar Zone was found to be poor. Knowledge about professional code of ethics, attitude towards professional code of ethics, and job satisfaction were significantly associated with the practice of code of ethics. The findings of our qualitative study also showed that lack of knowledge, unfavourable attitude, unfavourable working environment, and poor satisfaction were determinants of the practice of the code of ethics. Therefore, policymakers, hospital managers, and administrators should give more emphasis and work on health professionals through training and continuous evaluations.

## Data Availability

All the data were included in the study and for the sake of participants' confidentiality, it will be available upon a responsible request from the corresponding author.

## Abbreviations

ANRS: Amhara National Regional State
AOR: Adjusted Odds Ratio
COR: Crude Odds Ratio
EFMHACA: Ethiopian Food Medicine and Health Care Administration and Control Authority
ENA: Ethiopian Nursing Association
FHPEC: Federal Health Professional Ethics Committee
FMOH: Federal Ministry of Health
